# Compartment Syndrome of All Extremities in the Setting of COVID-19-Induced Systemic Capillary Leak Syndrome With Superimposed Myositis

**DOI:** 10.7759/cureus.41368

**Published:** 2023-07-04

**Authors:** Robert B Zeller, Casey Morehouse, Tom Lindsey, Aaron Provisor, Michael J Naylor

**Affiliations:** 1 Orthopaedic Surgery, Edward Via College of Osteopathic Medicine, Spartanburg, USA; 2 Surgery, Edward Via College of Osteopathic Medicine, Spartanburg, USA; 3 Simulation and Technology/Surgery, Edward Via College of Osteopathic Medicine, Spartansburg, USA; 4 Internal Medicine, Prisma Health, Sumter, USA; 5 Vascular Surgery, Prisma Health, Sumter, USA

**Keywords:** emergency fasciotomy, viral myositis, microvascular complications, bilateral compartment syndrome, systemic capillary leak syndrome, covid 19

## Abstract

Three years following the pandemic’s emergence, COVID-19 has continued to affect much of the symptomatic population with widely varied respiratory complaints, fevers, numerous unexpected prodromal manifestations, and unknown long-term consequences. Scattered cases involving myopathies, rhabdomyolysis, and compartment syndrome have also been reported throughout the pandemic. Some similar cases have been attributed to systemic capillary leak syndrome (SCLS). Here, we report the development of compartment syndrome involving all extremities in a 57-year-old vaccinated female known to have COVID-19. In retrospect, we believe the clinical severity and the patient’s sudden deterioration can also be attributed to the lesser-known SCLS. Treatment required fasciotomies of both forearms, arms, and legs. This is the most significantly involved case, leading to survival reported thus far. Lab abnormalities, misleading imaging, and symmetric involvement of all extremities posed a significant challenge to proper diagnosis and treatment. This case serves as a reminder for providers to remain cognizant of neurovascular emergencies during the workup of critically ill patients when the presentation is unrecognized and usual treatments are refractory. Its purpose is also to contribute to the global understanding of and response to COVID-19.

## Introduction

As the population shifts its concerns elsewhere, SARS-CoV-2, colloquially known as “COVID-19,” can still be found among our hospitalized patients and may appear as an incidental finding due to hospital screening requirements. Typical manifestations include fevers, coughing, dyspnea, myalgias, and significant fatigue. Loss of smell or taste, corneal congestion, diarrhea, and hypercoagulable states have also been observed; other rarer complaints involve the nervous, cardiovascular, and gastrointestinal systems [1]. Moreover, concerns are now about “long Covid” and the associated effects to be discovered for an unforeseen timeframe [2].

Myositis, rhabdomyolysis, and other rheumatologic symptoms have also been ascribed to COVID, but without as much attention relative to more common emergent presentations [3-5]. In a similar domain of musculoskeletal complications, acute compartment syndrome (ACS) in COVID patients has been rarely reported. The most recent case was written only a few months before the patient described here and is notable for appearing in multiple compartments [6]. Of note, this previous case was not linked to a specific pathophysiology.

Finally, a significant appreciation for microvascular changes in COVID-19 has begun to gain awareness [7-9]. Systemic capillary leak syndrome is a rare, often fatal, and sometimes chronic disease linked to microvascular changes due to systemic etiology [10,11]. There are an estimated 260 reported cases since its initial description. It is hallmarked by the “Three ‘H’s”: hypotension, hypoalbuminemia, and hemoconcentration. Mortality is high, and the presentation is known to appear much like septic shock, dehydration, primary polycythemia, and various other concerning presentations - leading many to believe it is underdiagnosed [11]. It may also lead to the development of compartment syndrome during the “extravasation phase,” particularly if there are excessive resuscitation efforts to avoid shock and multi-organ failure due to third spacing. There is no specific link between systemic capillary leak syndrome (SCLS) and COVID, though it has been seen with COVID previously by Ye et al., where it was attributed to the cytokine storm observed in severe COVID infections [12]. Two similar cases of SCLS, one involving a previously healthy patient and another patient with a history of SCLS who succumbed to a repeated episode after developing COVID, are well-described in a case series by Cheung et al. [11]. Similar case reports to Ye et al., such as that of Brod et al., may then be better explained in the underlying cause of the presentation [6]. The case described here will continue to expand on this virus’s role in SCLS in a patient experiencing prodromal symptoms such as other patients with myopathies and ACS whose treatment required fasciotomies in all extremities. 

## Case presentation

A 57-year-old female veteran arrives at the emergency department via emergency medical services complaining of viral symptoms, including generalized weakness, myalgias, and mild dyspnea for the previous two days, after testing positive for COVID-19 five days prior. She was vaccinated with two boosters but is now nine months overdue for a booster. She waited to come in today because of the increasing pain in her extremities. She was not in any respiratory distress, and her oxygen saturation remained >90% while on room air. It was decided to admit her to inpatient medicine for further evaluation, work-up, and initial treatment for dehydration with acute kidney injury (AKI). Given the nonspecific findings, the goal was to determine the primary etiology when her condition improved.

On exam, all extremities were symmetric, mildly edematous, and tense, with a painful active range of motion (ROM). Passive ROM was not initially painful. She remained neurovascularly intact (NVI) to the distal digits in all extremities, though baseline understanding of extremity appearance was difficult to ascertain. The remainder of the exam was unremarkable for her presentation.

Labs were consistent with dehydration despite administering two liters of lactated Ringer’s bolus (Table 1). Blood cultures were obtained without any growth after three days. PaCO2 and white blood cells were within normal limits, with repeated confirmatory labs, at 37 mmHg and 5.6 K/uL, respectively. Early rhabdomyolysis was suspected at this time based on clinical findings with rising serial CK results (Tables 1-2). JAK2 mutations were also considered for polycythemia vera (PCV), given the hemoglobin levels and systemic symptoms, but this lab result would not return for another two weeks. Deep vein thrombosis (DVT) prophylaxis protocol was initiated with 60 mg enoxaparin, with continued aggressive rehydration using lactated Ringer’s bolus and hydromorphone for pain management.

**Table 1:** Initial Workup Lab Results. Lab results reported at intake with units and normal ranges beside the name. Values reported with (H) or (L) indicate “abnormal,” either high or low, respectively, by hospital standards. Values reported with (HH) or (LL) indicate “critically” high or low, respectively, by hospital standards.

Value Measured	Reference Range	Value Amount
CBC		
WBC	3.5-10.8 K/uL	5.6
%Neutrophils	-	60.9
%Lymphocytes	-	23.2
RBC	3.86-5.35 M/uL	6.72 (H)
Hemoglobin	11.0-15.4 g/dL	19.0 (H)
Hematocrit	35.6-47.3 %	56.0 (H)
MCV	79.0-100.0 fL	83.3
MCH	23.7-32.9 pg	28.3
MCHC	30.0-36.5 g/dL	33.9
RDW-CV	11.6-16.0 %	12.7
RDW-SD	36.4-46.3 fL	37.6
Platelets	150-400 K/uL	249
MPV	9.2-12.8 fL	9.2
CMP (with additional related values)		
Sodium	136-145 mmol/L	120 (LL)
Potassium	3.5-5.1 mmol/L	5.6 (H)
Chloride	98-107 mmol/L	93 (L)
CO2	20-30 mmol/L	16 (L)
Anion Gap	6-16 mmol/L	11
BUN	7-20 mg/dL	22 (H)
Creatinine, Serum	0.57-1.11 mg/dL	0.87
Glucose	70-99 mg/dL	112 (H)
Calcium	8.5-10.4 mg/dL	7.7 (L)
AST	5-34 IU/L	66 (H)
ALT	<55 IU/L	17
Alkaline Phosphatase	40-150 U/L	54
Protein, Total	6.2-8.3 g/dL	5.0 (L)
Albumin	3.5-5.0 g/dL	2.1 (L)
Total Bilirubin	0.1-1.2 mg/dL	0.5
Phosphorus	2.3-4.7 mg/dL	4.8 (H)
Magnesium	1.6-2.6 mg/dL	1.9
Osmolality, Urine	50-1,200 mOsm/Kg H2O	927
eGFR	>59 mL/min/1.73 m2	78
Coagulation Panel		
PTT	24-36 seconds	32
Protime	11.9-14.3 seconds	13.6
INR	0.8-1.2	1
D-Dimer	<0.50 ug/mL FEU	1.33 (H)
Additional Reported Values		
CK, Total	29-168 IU/L	3,512 (H)
Lactate	0.5-2.2 mmol/L	4.1 (HH)
Sed. Rate (ESR)	0-30 mm/hr	12
Procalcitonin	<0.10 ng/mL	0.12 (H)
LDH	125-220 IU/L	397 (H)
Ferritin	5.0-204.0 ng/mL	173.2
CRP, Inflammatory	<=5.0 mg/L	13.0 (H)
TSH	0.350-4.940 uIU/mL	0.552
BNP	<100 pg/mL	40
Aldosterone	0.0-30.0 ng/dL	41.1 (H)
Cortisol	6-23 mcg/dL	18.5

**Figure 1:**
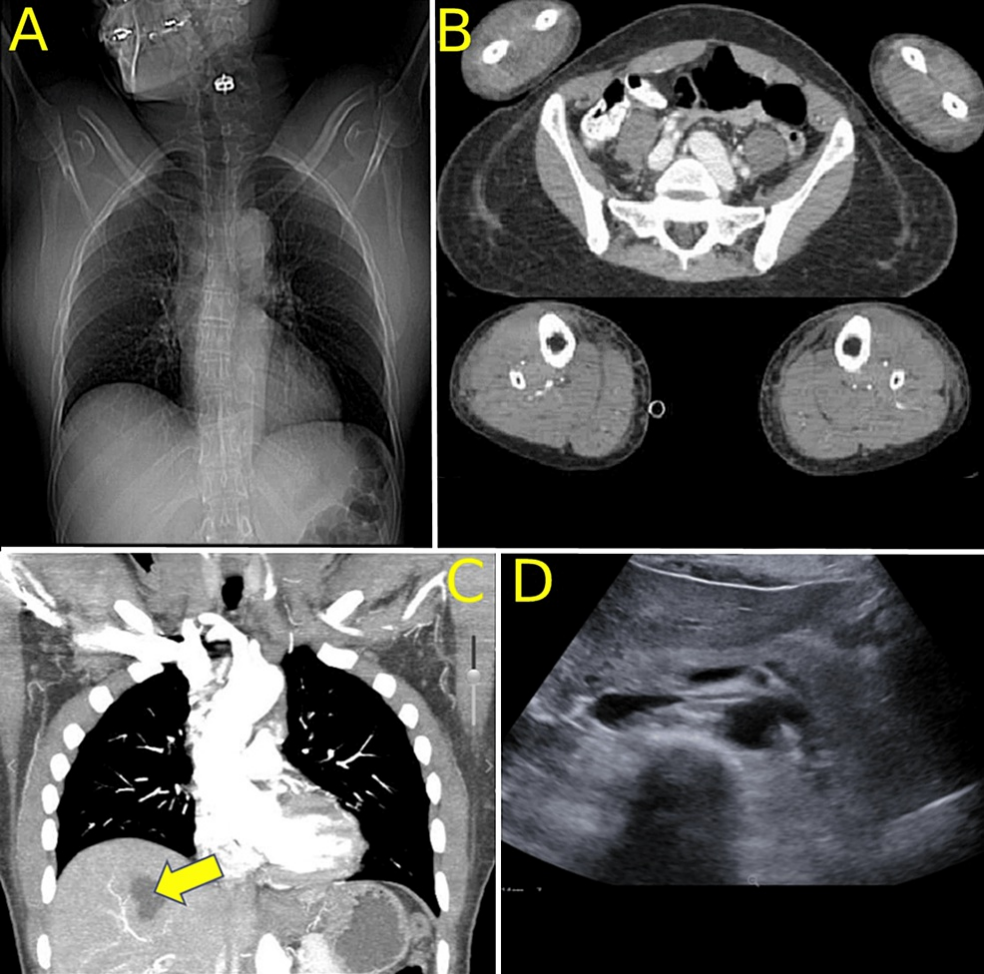
Initial Workup Imaging Results. Imaging obtained during her initial evaluation included: A) Anteroposterior radiograph obtained by CT, B) CTA of extremities (with runoff) in the axial plane to rule out vascular thromboses, C) CTA, in the coronal plane, which reported unknown “hepatic cysts,” with D) follow-up ultrasound of cysts, months later, showing a benign appearance. Additional imaging included vascular ultrasounds of all extremities to rule out thromboses, which were excluded due to the lack of information provided by a single frame. They were interpreted as showing nonspecific waveform abnormalities in the right upper extremity arterial Doppler US.

Imaging began with a chest X-ray showing nonspecific changes (Figure 1A). Computerized tomography angiography (CTA) of extremities was utilized to rule out arterial thromboses (Figure 1B). CTA of the chest, abdomen, and pelvis confirmed the findings of the chest x-ray. There were no pulmonary emboli. Incidental hepatic cysts were noted, which broadened the differential diagnosis and delayed workup (Table 1, Figure 1C). Venous ultrasound (US) of all extremities was negative for DVT. Arterial US of the upper extremities, where the pain was subjectively worse, showed mildly decreased flow with arterial waveform abnormalities. This was not considered relevant during the time of evaluation.

The following morning, the patient’s pain was also no longer tolerable with dihydromorphine, and she was in significant distress on the exam. Physical exam otherwise showed painful passive ROM in all extremities and mottling of the distal extremities, which was not noted on her initial intake. Nerve testing showed no sensation in the superficial peroneal distribution bilaterally. The sum of these features led to the decision to escalate care and evaluate for compartment syndrome emergently. Remdesivir and dexamethasone were administered, with the patient being admitted to the intensive care unit (ICU), as well as placement of a vascular consult for intracompartmental pressure readings. A Stryker STIC intracompartmental pressure monitor device was used to confirm forearm compartments were 48 and 65 mmHg in RUE volar and dorsal compartments, respectively, with 38 and 36 mmHg in the LUE volar and dorsal compartments, respectively. Leg compartments were presumed to approximate the upper extremities in pressures due to appearance, loss of sensation, and situational emergency. Morning labs later returned, showing a failure to improve overnight with creatine kinase (CK) elevated to over 23,000 IU/L (Table 2).

**Table 2:** Patient Fluctuating Lab Findings. SCLS-relevant labs obtained during the patient’s hospitalization are shown. The days provided are approximate and chosen to show the shift in appearance during each phase: from the initial labs with intake, followed by the same labs at ICU admission, and later during the recovery phase. It is worth noting that she is believed to have been experiencing the “prodromal phase” approximately two days before her presentation.

Patient Marker	Day 1	Day 3	Day 7
Blood Pressure (mmHg)	118/84	184/69	150/74
Hemoglobin (g/dL)/Hematocrit (%)	19/56	17.2/51.2	7.1/21.0
Albumin (g/dL)	2.6	1.8	1.3
Creatine Kinase (IU/L)	354	23,628	10,224
Pulse (bpm)	84	74	82
Temperature (°C)	36.6	34.7	36.9

The patient was taken to the operating room for standard fasciotomies involving the upper arms, forearms, legs, and all compartments therein. The thighs remained spared. All incisions were covered with gauze and ace bandage wrapping before returning to the ICU. Postoperatively, she remained intubated in the ICU with a central line in place. Over the next few days, she continued to receive aggressive care. Post-operative day three, she returned to the OR for wound debridement and dressing changes, and she remained intubated after, for continued wound management.

She required a complicated stay over the month following her transition into the recovery phase of SCLS, related to the development of Staphylococcus aureus pneumonia and a soft tissue infection in the right arm, which were without long-term consequence. She was subsequently discharged to a local rehabilitation and wound care facility, where she remained for another three weeks. Afterward, outpatient management involved four to six weeks of return to the local outpatient wound care facility before fully recovering.

## Discussion

During the COVID pandemic, medical attention has been focused on respiratory effects, but this case of SCLS due to COVID is an interesting example of immune responses to viral infection. For internal causes, systemic intracompartmental fluid shifts have underlying physiology involving hydrostatic pressure changes across capillary membranes, most often due to a much more obvious and chronic nature. Acutely, systemic fluid shifts are most often due to a signaling mechanism in response to something deemed "external" by the immune system, but the signaling in these cases has a more obvious or known mechanism at play, unlike that of SCLS. 

In the emergent scenario, the typical presentation of local compartment syndrome has a preceding incident with tissue trauma, and it is one of the most important considerations, leading it to be more easily recognized. In a patient with compartment syndrome in all extremities, without obvious massive trauma, there are limited options for possible diagnoses. Early signs of compartment syndrome involving multiple extremities, in the setting of hallmark SCLS findings, should lead clinicians to place SCLS high on the differential with a low threshold for ICU consultation for any changes in clinical condition.

In consideration of the literature, a few cases were particularly helpful for use in our retrospective diagnosis. In the pre-COVID era, two cases, both with the hallmark presentation, are worth noting here. The first involved multiple, recurrent episodes of idiopathic SCLS, with compartment syndrome and rhabdomyolysis, in a similar-aged, otherwise healthy male [13]. The patient's swelling began in the lower extremities, after a long flight, with a DVT being ruled out before the patient was sent home, only to return four days later, in approximately similar condition to our patient. These authors noted a retrospective recognition of a preceding episode of arm and leg pain with swelling, which would have served as a clue, had the clinicians known to consider this as the "transient ischemic attack" or "unstable angina" equivalent for SCLS when obtaining a history. For lab findings, this case was unique in that it had a monoclonal gammopathy of undetermined significance (MGUS) at the first episode of SCLS, but in a second episode of SCLS, months later, the serum protein electrophoresis (SPEP) was negative. In this second episode, the authors noted low C3 and C4 levels, although the diagnostic relevance of this is unknown. The second case involved an early 30s Hispanic laborer, with a record of hypertension and cocaine abuse, with similar prodromal symptoms beginning the day prior at work, aside from the addition of mild dizziness and confusion [14]. His case presented much more acutely, with compartment syndrome in all extremities, but involving the thighs in place of the legs. There is no mention of rhabdomyolysis considerations, labor intensity in the preceding day, or recent cocaine use. Both of these cases, although considered idiopathic, are important in that the leading differential diagnosis was PCV, prior to the recognition of a clinical emergency, which is not unlike our patient. In PCV, some of the mandatory diagnostic criteria do not result in a timely manner. This may be an important consideration in patients with an elevated hemoglobin finding, where it is worth establishing criteria for admission to monitor for SCLS over three to five days - to further elucidate abnormal findings and mitigate patient damage incurred. 

In the presentation of SCLS, the swelling may subtle due to a lack of fluids in the preceding days. With this, a detailed history of the preceding days is needed for monitoring, as timing is particularly important for the anticipated time until the extravasation phase. Wu et al. have previously discussed the autonomic biomarkers in SCLS, for consideration of targeted treatment, where they have noted the frequency of preceding "mild upper airway infections," suggesting its consideration as another key history component [15]. The initial workup for our patient was limited due to situational demands and delayed presentation. However, the provided medical history proved valuable, as it revealed that the patient was recently seen for an unrelated wrist MRI in the evaluation of a service-related injury, where screening had shown her to be COVID-positive. To ensure a comprehensive evaluation, it is still important to consider additional factors such as adequate hydration, changes in thirst, patient-observed skin changes, new or unique medication considerations, family history of autoimmune diseases, exposure to sick contacts, recent travel, dietary changes, and associated symptoms that extend beyond nonspecific viral prodrome, whenever clinically relevant and in the absence of other diagnostic clues.

In the first COVID-induced SCLS cases recognized, Case et al. identified a similar-aged male with primary hypertension, but otherwise healthy previously, whose presenting symptoms were more respiratory in nature [16]. Lab findings were strikingly similar, despite the respiratory symptoms, although they were more pronounced. Consistent with all of the similarly described cases in this case report, of any etiology, supportive care was initiated with aggressive fluid administration and the use of vasopressors. He ultimately developed compartment syndrome in all of the same compartments as our patient, with subsequent fasciotomies. Unfortunately, despite these, he continued to deteriorate prior to the family's decision to withdraw care. This case, much like our case, was described at the height of COVID-19's initial nationwide shutdown. It is unclear whether the use of remdesivir and dexamethasone or her use of vaccines played a role in outcome success in comparison, as immense progress and understanding of COVID-19 were made in the two years between these cases. 

**Figure 2:**
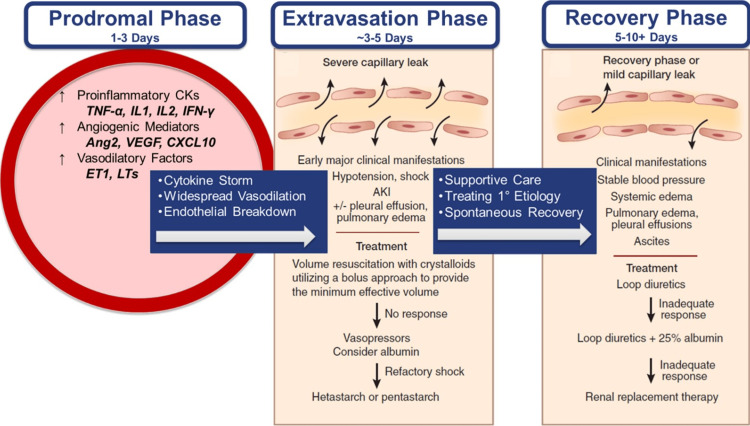
Pathophysiology of COVID-Induced SCLS. It has been observed that immune-mediated cytokine storms form in response to the virus, leading to angiogenic and vasodilatory responses, resulting in endothelial compromise. The widespread “third spacing” of plasma results in hemoconcentration during the extravasation phase, where the patient's clinical appearance shifts. Early diuresis during the recovery phase then allows for optimal recovery. Image adapted by the author (Zeller) from Siddall et al. [17], under CC license.

To understand the pathophysiology involved in SCLS due to COVID, Figure 2 was created to explain what is currently understood about SCLS [17]. Further, it is known that there is a Th1-skewed cytokine storm experienced in specific populations with COVID-19, which initiates the cascade required for SCLS to occur [12]. SCLS may be seen as a complication of sepsis or otherwise confused with sepsis due to the presentation similarity [6]. There are subtle differences that are key for treatment considerations and may explain the lack of recognition due to the poor outcomes which follow. In a similar fashion, the acute and immediate inflammatory response is much the same. What follows in SCLS, is its massive angiogenic and vasodilatory response, in particular, a distinguishing factor that leads to intractable hypotension and hemoconcentration. In addition, it has been suggested that there is a successful response to the "cholinergic anti-inflammatory pathway," which dampens the sympathetic response [15]. This is important in explaining our distributive shock that does not meet any of the systemic inflammatory response (SIRS) criteria (Figure 2). Other known links for SCLS include a response to autoimmune phenomena and certain immune-altering and chemotherapeutic medications (interleukins, monoclonal antibodies, and emtricitabine) [15,18]. It is unknown what may predispose a COVID-19 patient to experience SCLS; however, the years to come may unravel more through continued cases and COVID-19's persistence. 

 

In the information provided thus far, it is worth revisiting the final paragraph of the introduction, where cases such as that of Brod et al. may be adequately explained in the given context [6]. In their described patient, an vaccinated, obese, early-30s female had been seen with a non-specific viral prodrome, including mild upper respiratory symptoms. She was discharged with instructions on supportive care and quarantine protocols. She began to develop fevers and leg pain in the following 24 hours, which led her to go to the ED for evaluation. We believe that this presentation would approximate one or two days prior to the case we have described. Her mild hypotension resolved with a crystalloid discharge, and she was discharged after duplex US and CT to rule out DVT or pulmonary embolus. The following day, she returned to the ED again, this time with intractable hypotension and hemoconcentration, which was mistakenly attributed to septic shock due to associated leukocytosis and tachycardia, but no fever and no mention of respiratory status. Treatments initiated were similar to all other prior efforts, with the same outcome of compartment syndrome, although isolated to bilateral legs. The surgical team involved managed to obtain intraoperative photos showing necrotic muscle, with muscle samples obtained to confirm the presence of myositis, but there was no mention of CK levels or rhabdomyolysis. This patient managed to make a full recovery due to prompt recognition and fasciotomy. 

**Figure 3:**
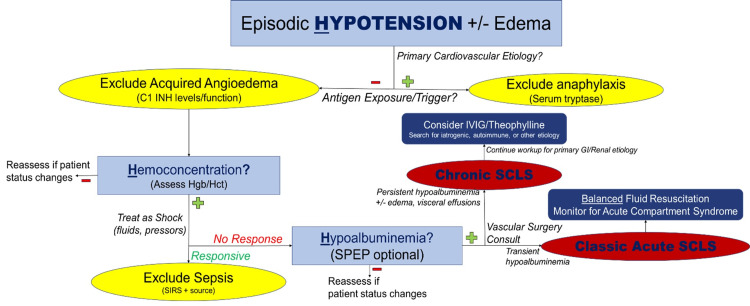
Approach to SCLS Diagnosis. The following algorithm has been proposed to assist in making the diagnosis. Note the three key steps involving the hallmark 3 “H”s of SCLS (hypotension, hemoconcentration, hypoalbuminemia). Image credits: Author (Zeller), updated with the most recent literature.

Presently, there is no ideal algorithm in the workup for SCLS, and it is a diagnosis of exclusion (Figure 3). Depending on the timing of the initial evaluation and information gathered, the differential diagnosis should be broad, including sepsis, PCV, adrenal insufficiency, amyloidosis, toxic shock syndrome, heart failure, engraftment syndrome, differentiation syndrome, ovarian hyperstimulation syndrome, hemophagocytic lymphohistiocytosis, viral hemorrhagic fevers, snakebite envenomation, ricin poisoning, or any other causes of "3rd spacing" or swelling without obvious signs or symptoms [17]. In the use of our algorithm, with no findings suggestive of an alternative diagnosis, hallmark findings of Table 2, and acknowledgment of the pathophysiology provided - we have arrived at the given diagnosis for our patient.

**Figure 4:**
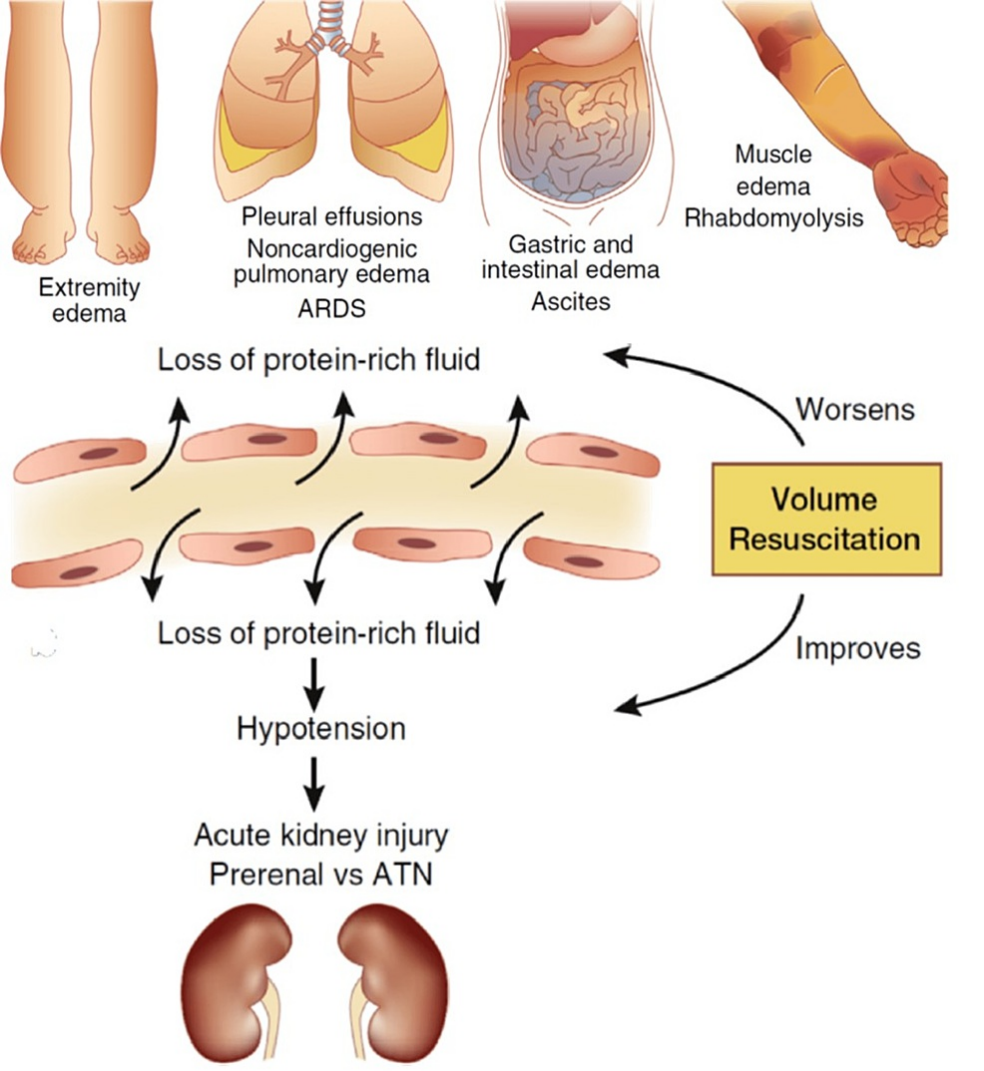
Fluid Resuscitation During Extravasation. Recognition of the condition typically occurs in the extravasation phase. This is when the endothelium permits uncontrolled fluid losses. Fluids must be administered carefully to avoid complications of excessive or unfruitful resuscitation. Image adapted by the author (Zeller) from Siddall et al. [17]*, *under CC license.

The extravasation phase of SCLS is typically when recognition becomes possible, as the prodromal phase presents similarly to several viral illnesses [17]. During this stage, there is no exact known way to treat SCLS. In addition, there is a very delicate balance to resuscitation (Figure 4) [19]. Overcorrection will preserve the cardiovascular system and compromise the areas of third spacing, and compartment syndrome, while under-resuscitation, leads to death due to shock and multi-organ failure. The compartment syndrome, while iatrogenic, will preserve life acutely, if closely observed for fasciotomy, but it is not an optimal circumstance. In revisiting the COVID-induced SCLS case series reported by Cheunget al., the clinician's astute, prompt recognition of SCLS, is one of the rare scenarios of early recognition, leading to a very thorough evaluation and description [11]. This awareness may have skewed the management in favor of conservative fluids, which successfully avoided compartment syndrome. These patients were given approximately 1/2 to 1/3 of the amount of crystalloid fluids, which were given to the cases of SCLS, which lead to compartment syndrome. Whether more aggressive fluid administration would have altered the outcome is hard to say, as these cases occurred around the time of vaccine release, when much less was understood. 

The prognosis in SCLS is dismal due to its ability to mimic other presentations and the fluctuating patient status, on top of potentially forcing clinicians to choose neither the pressure in the operating room for emergency surgery or their death. SCLS prognosis worsens by its ability to be recurrent in some populations, as described in [10,11]. The primary goal then currently revolves around reactive treatment upon recognition, close monitoring for a change in condition, and diuresis during the recovery phase; additionally, anti-inflammatory therapies are currently of great interest to researchers [17,18]. Anecdotally, theophylline, beta-2 adrenergic agonists, and even imatinib have been used to promote vascular integrity with early recognition in the acute scenario [18]. In those who have recurrent attacks, theophylline and IVIG (2 g/kg) have shown some success for prophylaxis. However, at present, there are no data to suggest the use of any proven treatments beyond supportive care and close monitoring for clinical changes. 

While this is not the first case of COVID-induced SCLS, nor the first development of ACS in all extremities due to SCLS, we are thankful that this was the first occurrence of both, in which the outcome resulted in the complete recovery of the patient, who has since been discharged from wound care and follow-up with the vascular surgeon. With this being a retrospective diagnosis, there are other considerations that we wish to have investigated. Though not mandatory for the diagnosis, we would like to have obtained evidence of monoclonal gammopathy of undetermined significance (MGUS), which appears to be sensitive for SCLS [18] (Figure 3). It is seen in 85-95% of SCLS cases by SPEP, but not unrelated to the rate of progression to multiple myeloma in other MGUS patients. In a similar manner, additional evidence for/against C3 and C4 diagnostic relevance is worth consideration. In an unrelated area, ACE2 receptors have consistently been a massive topic for treatment discussions in treatment for COVID-19, due to its use in cell entry [7]. Further, a subsection of this research was investigating links and similarities between sarcoidosis and COVID-19 [20]. We would like to have evaluated this further in her workup, as many radiographs were obtained to evaluate the presence of COVID pneumonia, with mild bilateral hilar lymphadenopathy being observed consistently (Figure 1A). This had unknown relevance and significance in this case due to unclear links and mild appearance on imaging. Despite this, we cannot rule out the diagnosis, as symmetric and bilateral hilar lymphadenopathy is a known sensitive finding in sarcoidosis. We chose to include this topic of discussion for the purpose of adding an additional possible link, within a larger area of microvascular pathophysiology and immune-related disease. 

## Conclusions

The COVID-19 pandemic has been seen with immense fear and controversy in society, and it remains not fully understood, with skepticism toward the scientific and medical community. This case is reported in the hopes that both the scientific community and the public will continue to understand this disease’s potential impacts better. COVID’s microvascular alterations, amid other complications, should remain on the differential diagnosis for an unknown future.

Further studies will also be of interest to observe similar cases and identify potential markers or imaging useful in screening populations at risk for SCLS or other microvascular events. In our case, the arterial changes on Doppler US may be worth investigating further, for potentially specific and consistent waveform changes, as a potential screening protocol in the workup of SCLS during the late prodromal phase; however, its presence among SCLS patients has not been described elsewhere thus far. 
